# Relational ambivalence: Exploring the social and discursive dimensions of ambivalence—The case of Turkish aging labor migrants

**DOI:** 10.1177/0020715219832918

**Published:** 2019-03-07

**Authors:** Monika Palmberger

**Affiliations:** University of Vienna, Austria; University of Leuven, Belgium

**Keywords:** Aging, ambivalence, Austria, belonging, home, labor migration, older migrants, relational ambivalence, Turkey, Vienna

## Abstract

Many of Vienna’s labor migrants who entered Austria as so-called “guest workers” together with their spouses long nurtured the dream of returning to their country of origin, at the latest when they retired. By then, however, returning became less than straightforward leading to ambivalence regarding questions of belonging/return and transnational mobility and late-life care. Based on rich qualitative data, in this article, I show that ambivalences are found in the complexity of migrants’ narratives, particularly in the way they (1) reassess past choices, (2) negotiate feelings of belonging, and (3) assess future options for late life and care. I argue that the social dimension of ambivalence, which I term “relational ambivalence,” is crucial to understanding the labor migrants’ experiences, reflections, and choices. The analysis shows that ambivalence must be understood as a product of relationships rather than solely an individual experience. The concept of relational ambivalence captures these social and discursive dimensions of ambivalence. The article ultimately carves out the particularity of ambivalence in the general context of migration and in the specific context of Vienna’s labor migrants, while accepting feelings of ambivalence or the simultaneity of different, opposing positions in one and the same person as a core human experience.

## Introduction

While social research acknowledges inconsistencies and contradictions as part of the complexity of human thought and practice, ambivalence is rarely the center of attention in the social sciences. With this in mind, David Berliner (2016) makes a plea for a “science of contradiction” and argues that “it is time to bring back ambivalent statements, contradictory attitudes, incompatible values, and emotional internal clashes as research objects” (Berliner, 2016: 5–6). While the migration context is particularly prone to producing feelings of ambivalence, as shown in this article, ambivalences, or the simultaneity of different, opposing positions in one and the same person need to be recognized as a core human experience. This means that while migrants’ particular lifeworlds are “conducive to feelings of ambivalence” (Kivisto and La Vecchia-Mikkola, 2013: 214), such feelings are not exclusive to them. Rather, they are an expression of the complexity of social life, which is filled with incompletion, contradictions, and ambivalences (see Jovanovic, 2016; Lambek, 2016).

In this article, I analyze the narratives of aging Turkish labor migrants in Vienna and their confrontations with sometimes seemingly irresolvable life decisions. Ambivalence came to the fore in the narratives of Turkish first-generation migrants, which reveal diverse feelings, experiences, and voices. Labor and hardship have defined life for these labor migrants, and it is in retirement that former so-called “guest workers” (*Gastarbeiter*) can pause to reflect on their past lives and future prospects. In this article, I show that ambivalences are found in the complexity of migrants’ narratives, particularly in the way they (1) reassess past choices, (2) negotiate feelings of belonging, and (3) assess future options for late life and care. I argue that the social dimension of ambivalence, which I term “relational ambivalence,” is crucial to understanding the labor migrants’ experiences, reflections, and choices.

## Ambivalence as an analytical focus

In scholarly debates, “ambivalence” refers to different states and processes. First, we can describe it as “simultaneous and contradictory attitudes or feelings (such as attraction and repulsion) toward an object, person, or action.” Second, we can describe it as “continual fluctuation (as between one thing and its opposite)” or as “uncertainty as to which approach to follow.”^1^ In this article, ambivalence too has multiple faces and is most often related to an experience/assessment of a particular situation (past and present) or to a decision-making process (see Merton, 1976; Smelser, 1998). The concept of “relational ambivalence” which I introduce in this article, highlights the relational and emotional aspects of ambivalence. It shows how assessments of particular situations and decision-making processes are made in conversation with several voices (particularly of family members). Thus, ambivalence is understood as a relational experience that is often tied to strong (relationally driven) emotions. Unlike sociological studies of migrants’ ambivalence that often focus on the ambivalence resulting from the different statuses and roles migrants inhabit in their country of origin and in their host country (Connidis and McMullin, 2002; Merton, 1976), this study reveals ambivalences as an outcome of migrants’ intimate relations.

Sociologists have been skeptical about the inclusion of the notion of ambivalence in their repertoire because it was associated with the fields of psychology and psychoanalysis where the phenomenon was studied as an isolated individual experience, thus paying too little attention to its cultural and social embeddedness (Hillcoat-Nalletamby and Philipps, 2011: 202). This changed with the recently emerging field of the sociology of emotions, thanks to which the notion of ambivalence finally became a legitimate research subject (see Weigert, 1991). One field that greatly benefited from this “opening” is the sociological and anthropological study of kin/family relations, in which the phenomenon of ambivalence is now central (Peletz, 2001: 426). The reason ambivalence and mixed emotions are seen as so crucial in kinship/family studies is that kin relations are tightly entangled with moral entailments, expectations, and obligations. As Peletz (2001: 434) argues, “ambivalence is a feature of virtually all kinship systems” due to the “contradictory structural imperatives inherent in such systems (and all other institutions) something like prescriptive amity or ‘diffuse, enduring solidarity.’” Mixed emotions thus often characterize personal bonds and are likely to be negotiated continually over the life course (Connidis and McMullin, 2002).

New kinship studies engage in critical analysis of power, agency, and sociality with a special focus on gender relations. Here, the work of Arlie Hochschild (Hochschild, 1997) on “emotion work” is particularly important and sheds light on the political, cultural, and economic contexts of ambivalence (p. 210). Hochschild (1983) shows that the “deeper the bond, the more emotion work is at place and the more unconscious we are of it” (p. 68). She also shows how emotion work is likely to be associated with ambivalent feelings and that there are cultural differences that define which are accepted and can be expressed and which not. While in my article, I do not deal directly with the mixed emotions that are likely to characterize strong emotional bonds, ambivalent feelings are discussed as social and interpersonal experiences that cause people to consider different viewpoints and emotional stances of close family members when rethinking the past and when making choices for the future.

In sociological discussion, ambivalence, however, goes beyond family/kin relations and is also discussed as a phenomenon characterizing the transition of modernity to post-modernity or what is sometimes referred to as “late modernity” (Bauman, 1990; Giddens, 1994; Smart, 1999). In these sociological discussions, ambivalence is said to characterize society, not least due to increasing social fragmentation and the loosening of social roles. This goes hand in hand with a fragmentation of lives and difficulty in foreseeing one’s life course due to social insecurities, short work contracts, the demand of increased mobility, and the need to “reinvent” oneself. Migrants are particularly challenged in this respect. In the work presented here, I analyze ambivalences as experienced by individuals within their particular family context but also embedded in wider social relations and specific cultural and historical contexts.

When my research participants (re)assessed past choices and reflected on their present lives and future options, this was done in exchanges with other family members whose emotionally informed positions were taken into account. Relational ambivalence, which I discuss in this article, is greatly informed by emotions, by the speakers’ emotions, as well as by emotions expressed by those close to them. I follow the path of scholars who argue that emotions are not a purely private matter, but are also communicated to and shared with others (see Abu-Lughod and Lutz, 1990; Boccagni and Baldassar, 2015; Leavitt, 1996). Moreover, emotions here are not understood as the antithesis of reason but as central to it and so are crucial in any decision-making process. We need to keep in mind that without emotions, social life, including our decision-making capacities and our ability to make informed choices among a plurality of options, would be impossible (Williams, 2009: 150). This also implies that emotions (expressed and shared with others) are understood not as an internal state but as part of social life. Therefore, the focus in this work is more on the social dimension of emotions without denying the force of emotions as subjective experiences (see Abu-Lughod and Lutz, 1990).

Not only emotions but also the notion of ambivalence itself has been revealed in my research as intrinsically social. Researching ambivalence in the context of old age and family solidarity, Hillcoat-Nalletamby and Philipps (2011) argue that “ambivalence cannot be reduced to an individual experience, disembodied from the wider web of interdependent social relationships to which it belongs and the social environments within which it is embedded” (p. 212). Such a relational approach also adds a temporal and transformative dimension to ambivalence and situates itself at the interface of individual experience and group belonging (Hillcoat-Nalletamby and Philipps, 2011: 214).

While ambivalence was not the focus of my research in the first instance, it emerged as a key category during inductive analysis. Kierans and Bell (2017) propose to “cultivate an analytic of ambivalence” and to focus on ambivalence as a methodological heuristic. I agree with them when they argue that

ambivalence, by definition, presents us with an important methodological paradox. While it foregrounds polarized positions, their apparent clarity provides little help in dealing with the complexities of the social situation at hand, complexities which can only be understood when we resist the choice between positions which seek to deny them. (Kierans and Bell, 2017: 36)

This also means, as Kierans and Bell (2017) argue, that we have to accept that “things are not readily clear” and to “explicate phenomena” in our analysis.

In my study, ambivalence emerged as a category that allowed a better and deeper understanding of the narratives aging labor migrants shared with me, since ambivalence expresses more than simple contradictions. First of all, my interlocutors did not express contradictory attitudes or feelings but the ambivalence they expressed rather resulted from the multi-voicedness, from the various angles they contemplated on a particular subject, and the views of different family members they took into account. This “relational ambivalence” I encountered in the narratives of aging Turkish labor migrants often had an implicit character. Meaning that if contradictions evolved in the narratives they shared with me, these were not presented as contradictions. Ambivalence thus was closer to indecision or to the acceptance of different standpoints/positions than to a contradiction as such. Even if, in our time of increasing pluralistic complexity, indecision expressed by individuals is easily branded as a fault, a weak ego, there are good reasons to interpret it otherwise. Weigert (1991), for example, suggests,

ambivalence can also be interpreted differently, as confidence to confront to both sides of an issue; ability to weigh alternative points of view and give each its probability; and strength to admit that there is no morally certain line of action. (p. 22)

Sometimes ambivalence in the narratives of my interlocutors also expressed a form of deferral of judgment or decision. Ambivalence also differs from ambiguity, especially when we understand the latter as a more cognitive (than emotional) phenomenon (Peletz, 2001: 415). Still, as I will show, the undecidedness and the acceptance of irresolvable life decisions did not put people in a passive position but ultimately proved itself to be a form of agency.

## Research contextualization and methods

The field of “aging and migration” only started to establish itself as an important research field in the early 2000s (see Hromadzic and Palmberger, 2018). One of its core research strands is the study of migrants who are aging in a country other than that of their origin. In Europe, this mainly concerns former labor migrants from Southern and South-Eastern Europe and Northern and Western European countries, such as Austria (see Ciubanu and Nedelcu, 2019; Karl and Torres, 2016; Palmberger and Hromadzic, 2018).

Austria and Turkey signed the first guest worker contracts in 1964. Of the approximately 265,000 people Austria recruited as guest workers (many more entered the country through other channels) in the period between 1961 and 1973, about two-thirds came from Yugoslavia and about one-third from Turkey. A significant number of these “guest workers” remained in Austria with their family members following them (Reinprecht, 2006; Von Lorber, 2017). When these migrants entered Austria as guest workers, they were given restricted work and residence permits and only temporary and sub-standard housing and were offered no integration measures (e.g. language courses). Austria recruited mostly unskilled workers for the construction industry but also for other sectors, including the textile and paper industries, and for the tourism business. As the capital city, Vienna attracted the majority of so-called guest workers, and thus offers specific opportunity structures, including a relatively high number of migrant associations and mosques (Palmberger, 2016). These labor migrants, male and female, significantly contributed not only to Austria’s post-WWII economic success but also to the diversification of Vienna’s cityscape. In many Viennese neighborhoods, this is evident in an ever-increasing array of shops, food stalls, hairdressers, cafes, restaurants, and so on owned by migrants offering a wide variety of products and services. As Glick Schiller and Caglar (2011) rightly argue, it is time to “recognize migrants as active participants in the reconstruction of urban life” (p. 196). Labor migrants from former Yugoslavia and Turkey were an important driving force behind this urban reconstruction—a fact rarely acknowledged.

Bad working conditions and resultant health issues forced many of the former guest workers to seek early retirement (Reinprecht, 2006). Due to this fact, the discussion here considers migrants of 55 years and older. All of them were still able to fulfill daily duties and were not in need for care. My interlocutors (or their spouses) were recently retired or in the process of seeking retirement. They thus shared a specific stage in their life, one in which they were retreating from working life, voluntarily or not. The exact age is therefore less important for this study than the stage of life people find themselves in (see Cohen, 1994; Keith, 1980). In addition to sharing the same cohort and migration history, my research participants shared certain socio-economic characteristics such as a low level of education, a low income/pension, and most often a rural birthplace.

These Turkish first-generation migrants came to Austria as “guest workers” or as their accompanying spouses. The majority of my Turkish male research participants came to Austria as labor migrants in the 1960s and 1970s and served as unskilled workers in Austria under precarious conditions. Their wives and children often followed at a somewhat later stage. The majority of my female research participants belonged to this group of people who came to Austria in the context of family reunion. Some of the female migrants I interviewed worked in the cleaning and catering industries after their arrival, two women worked in home care, and the others did not enter the labor market at all.

Although the family context varied, all of my research participants were married (one widowed) and had children and in most cases also grandchildren. Many of the children and all grandchildren were born in Austria. All of my research participants had close family members in Austria, meaning that they had at least a spouse or children in Vienna. More than half of my research participants were Austrian citizens, more of whom were men than women. For those who were not citizens, the bureaucratic procedures and particularly the German language test one has to pass today in order to gain Austrian citizenship constituted a hurdle too big to overcome.

As noted, many of my research participants had only a basic education. The highest level of completed education among my male interviewees was an apprenticeship or an equivalent thereof, but many only completed primary and sometimes some years of secondary school. My female interviewees generally had a lower level of education and many had only attended primary school for a couple of years; some had no school education whatsoever.

While German language skills varied, men I talked to tended to speak German better than the women. This is connected to the fact that men were more fully integrated into working life, and thus were confronted more often than women with the German language. Many of my interlocutors, particularly women, told me how much they regretted not having had the chance to learn German properly. They said that if they had the opportunity to come to Austria again, they would focus their energy on learning German, and some of those who had not worked added that they would seek work if given a second chance.

This article discusses research based on a wider ethnographic study that examines social relations and ideas of transnational aging and care among older migrants from Turkey and from former Yugoslavia in Vienna. The specific part of the research with Turkish labor migrants I present here is primarily based on 28 semi-structured narrative interviews conducted between 2013 and 2016 with 25 interviewees (each lasting between 45 minutes and 2 hours) and is further informed by informal interviews (30), expert interviews (11), and participant observations. The gender division of the 25 Turkish migrants with whom I conducted in-depth interviews was almost equal, with 13 women, and 12 men. I asked questions that invited them to examine their present life by connecting it to past traits and future aspirations. The interviews thus give insight into past decisions, present life strategies and concerns, as well as into hopes and fears regarding the future.

I remained in contact with several of my interview partners after the initial interview. I have been a guest in their houses where I met and spoke with other family members, including spouses, children, and grandchildren. With three of these key research participants, I conducted a second interview round between 1 and 2 years after the first interview. These gave me the opportunity to delve deeper into selected topics and to hear how certain decisions for late life have developed since the first interview.

I established contact with interviewees personally or through different cultural, religious, and political Turkish associations. My two research assistants themselves had Turkish roots and were central in initiating first contacts. It was important for the wider study that my interviewees should differ in their political/ethnic/religious positionalities, and the different family backgrounds and life contexts of my two research assistants made it possible to make contact with practising Sunni Muslims, Alevi Muslims, as well as atheists.

I carried out interviews either in the interviewees’ homes or in one of the migrant associations of which they were members. I conducted interviews in the language interviewees’ preferred—either German or Turkish and recorded and transcribed them for reasons of storage and analysis. Those in Turkish were conducted together with my research assistants and were fully translated. The interview citations in this article are all English translations. I analyzed the interviews by coding and developing categories according to grounded theory (Kelle, 2007; Palmberger and Gingrich, 2013).

## Labor migrants after work: reassessing past choices

The narratives people shared with me are individual accounts but also narratives of Europe’s labor migration in the 1960s and 1970s during a time of reconstruction and economic recovery after WWII. Most of the labor migrants and their spouses I talked to migrated because they were in need of jobs and/or because they expected a job in Austria to pay better and to guarantee their family some form of social advancement. They trusted that this move would enable them to provide a better future for their children, and a better education leading to a qualified job. This said, they experienced multiple hardships, including poor living conditions. They mostly came from rural Turkey where they owned their houses, but in Vienna they had to live in small sub-standard accommodation often with no running water inside and only shared sanitary facilities outside on the communal staircases.

The labor migrants’ children, the second generation, appreciated the sacrifices their parents made but at the same time were aware that their parents’ quality of life and health had suffered tremendously; they often used the phrase *kaputt arbeiten* (working hard for a living) in this context. Furthermore, their parents’ expectations weighed heavily on them.

With this goal of achieving a better future for their children (and grandchildren) as well as upward social mobility, on retirement aging labor migrants reassessed whether migrating to Austria was the right decision. Some were able to reflect on how successful their children had become and how they had gained a higher education and a respectable job. This was the case for Orhan, who worked as a painter in Vienna for more than 25 years and was recently retired:

It is difficult for me to say … of course it was the right decision to come here; I came as a simple house painter. And maybe back then I could not have dreamt of sending my daughter to the University of Vienna, that a painter could send his daughter to the University of Vienna! After all, only a billionaire can do that in Turkey, right? […] I like this country; I have no problems with it. I only have a problem with its politicians …

As became apparent later in the interview, which I discuss in more detail below, despite the opportunities, Orhan could give his children in Austria, and though he was very grateful that they had a good education, he was ambivalent toward Austria and felt that he did not fully belong there or, more precisely, that he was not granted full citizenship. Orhan told me, “They give us Austrian passports but during elections you are confronted with racist parties’ posters; they use offensive words to make anti-Turkish propaganda.”

Unlike Orhan’s children, many of the other labor migrants’ children experienced difficulties at school and with integrating into Austria’s job market. The difficulties associated with bringing children to a new place where one does not speak the language and providing them with a good education and prospects were central for many of my interviewees when reassessing past choices. In the following, I will illustrate with Bahar’s story how the decision to migrate is reflected upon critically and stirs ambivalent feelings in late life. I visited Bahar several times, conducting two narrative interviews with her in her home, where I also had the chance to meet and talk to her husband and two of her children and to meet three of her grandchildren. In her narrative, the discrepancies between the idea of achieving a better life in Vienna, with all its promise, and the hard life Bahar and particularly her children experienced once in Vienna are most prominent and cause her ambivalent feelings toward Austria.

Bahar’s doubt that it was the right decision to come to Vienna results most strongly from her frustration with the inadequate education her children received in Austria; she believes that her children, especially the two eldest (who were already 10 and 11 years old when they moved to Austria), would have received a better education had they remained in Turkey. Bahar’s father took her out of primary school during her first year, and it has always been her heart’s desire to guarantee a good education for her offspring, something she herself was never granted even though she longed for it. Indeed, the two older children were eventually sent to a special school (*Sonderschule*), a common practice affecting many children of “guest workers” due to their (and their parents’) poor German skills. For these reasons, she is unsure if it was the right decision to follow her husband to Vienna together with her children:

If I’d remained in Turkey, then my children would have received a better education; they would have reached a higher level … My children were smart; my daughter in particular was extremely clever … she became a hairdresser, but she could easily have been a medical doctor; she was a very smart kid! With hindsight, I think it would have been better if my husband had come to Vienna alone and I’d stayed with the children in Turkey and they had received their education there … It is only because those [Turkish labor migrants] who returned always bragged [about their life in Vienna ] and so we thought it was something special.

This quote makes clear how high the expectations and dreams of a better future were among labor migrants, expectations fed by those who already lived in Austria, and on their return spread the “myth of prosperity.” Bahar moved from a house in rural Turkey to a one-room flat in Vienna without running water and with a toilet outside, and she describes the first years after arrival as particularly burdensome. Nothing was as comfortable as she had expected but she hoped she would eventually prosper. By the time Bahar recognized that this was a myth and a longing that would never be satisfied, it was too late, and this is very difficult for Bahar to accept. The fact that she perceives herself as the main person responsible for the wellbeing of the children makes the realization that Austria has not fulfilled what it seemed to promise particularly painful, especially in regard to social advancement.

At several points when Bahar assessed her past decision to migrate to Vienna and when she talked about the impossibility of leaving the city, her narrative shifted into a form of inner dialogue. Her words no longer seemed to be addressed to me, the interviewer, but rather to herself. This strengthened the impression that the tensions about past choices were present for Bahar not only when she expressed herself during the interview process but also that they had been troubling her for some time.

Bahar’s ambivalence toward Austria is fed by the lack of (educational) opportunities her children received there. This specific disappointment contrasts with her otherwise positive feelings toward Austria, as I will discuss in more detail in the next section. For Orhan, whose children went into higher education, the situation differs. As shown, his ambivalence toward Austria stems, on the one hand, from the good opportunities it afforded his children and, on the other hand, the political developments there that he assesses critically and the increasing xenophobia/Islamophobia he is confronted with.

Concluding this section, we can say that the ambivalence my research participants felt when reassessing past choices was closely connected to the lives—including the opportunities and challenges—of family members. Bahar’s ambivalent feelings concerning her decision to migrate to Vienna arise from her children’s experiences of discrimination in Austria’s school system and these often overshadow her good memories of past times in Vienna. For Orhan too, the decision to migrate to Vienna is ambivalent but for other reasons. While he considers the educational and professional achievements of his children as a big success, his personal experiences in Austria are far less positive. While Bahar’s and Orhan’s experiences differ greatly, they both take into account other family members’ experiences when assessing past choices. The social dimension of ambivalence is foregrounded, and it is a relational ambivalence we encounter here.

## Negotiating home and belonging: ambivalent nostalgia

Ambivalence also came to the fore when negotiating present feelings of home and belonging. The phase around retirement was a significant time in the life course of my research participants, when they not only reassessed the past but also reflected on questions of belonging. It was an important time for men, who in most cases provided the family with the main income, as well as for women, even if many of them were not integrated into Austria’s job market or were only employed part-time. Regardless of their employment status, life also changed for them when their husbands retired and their children moved out. My interviewees’ decision-making process, especially regarding where to spend late life, was rarely easy and straightforward but more often than not was characterized by ambivalent feelings, as I will discuss in more detail in the section on late-life care.

Home for my research participants held a plurality of meanings and was not a fixed category but rather was free to be negotiated (see Bilecen, 2017; Ehrkamp, 2005; Uehling, 2002; Wong, 2002). While feelings of home regarding Turkey were closely linked to it being the place of “origin,” childhood and socialization, they experienced Vienna as home mainly due to present social relations and as the place where family life took place and where friendship and community life were nourished on an everyday basis (Palmberger, 2018). As other authors point out (see, for example, Bolzman et al., 2017; Ganga, 2006), it is not the privilege of elite migrants to claim both countries as their own and split their time between the two. Ganga (2006) describes a similar situation of constant commuting between two countries for Italian labor migrants in the United Kingdom and how they “move freely in spaces they feel belong to them. In fact, whether they feel fully integrated or not, they oscillate between the two societies, being members of both” (Ganga, 2006: 1406; see also Boccagni, 2012, 2017).

Previous research concentrating on aging migrants, and in particular on Europe’s Turkish labor migrants, highlighted the transnational dimension of their lives, characterized by multiple or “altering” residence (see Baykara-Krumme, 2014). Even those whose main residence is in their European destination country maintain strong transnational ties. “Living” in the two countries not only takes place through regular commutes but is also a much more complex process of fostering transnational ties. This includes what Bilecen et al. (2015) refer to as “stitching”: “a process in which the migrants in the host country continuously attach themselves to the society back in their country of origin” (p. 254). Thereby migrants’ identities “cut across fixed notions of belonging” (Dwyer, 2000: 475). Yeoh and Huang (2000) discuss this phenomenon as a constant maneuvering to reconnect and disconnect with home, and Vertovec (2004) as dual-orientation or “bifocality.” While most of my interlocutors maintained close relations to both countries and finding transnational spaces for themselves, the decision about which country to choose as the main residence after retirement was not always easy, as exemplified by the case of Ahmet. When I asked Ahmet about where he and his wife plan to spend late life, he answered,

That is a big question mark … mostly here, not in Turkey. […] For example next week we are going to Turkey on holiday for two-and-a-half-months at the most but then we’ll come back. This is unfortunate … There [in Turkey] is my country, but my life was here [in Vienna], that’s why. Moreover, my children are here … in three months we have two big religious holidays, then we have to be with our children …

Ahmet’s answer reveals ambivalent feelings concerning belonging. The statement “there [in Turkey] is my country, but my life was here [in Vienna]” is particularly telling. It implies that the place where Ahmet has lived most of his life has not become “his country.” Still, there is no either/or option available to him. Both countries have become meaningful to him, although for different reasons—Vienna because it is the place where he and his wife have spent most of their lives, where their children were born and which they now identify as their country, and Turkey because it is the place Ahmet was raised and socialized and because it has remained his emotional home ever since. We could also say that home is split into “the place of origin” and “home as the sensory world of everyday experience” (Ahmed, 1999).

Those who opted for dual-residency often said that they felt constantly homesick because they always miss one of their homes, either Austria or Turkey. As one of my female research participants put it: “When we’re here in Vienna we miss Turkey a lot. But when we’re in Turkey during the summer months we really miss Austria!” They expressed such feelings of home and (be)longing, however, not only in relation to present-day Austria and Turkey but also toward a third home, located in the past. However, this third, temporal home located in the past did not always have clear geographical boundaries, and often it was not obvious whether the past in Turkey or Austria was being referred to. The past then was first and foremost set in juxtaposition to life in the present—the past home being characterized by, for example, “trust” and “moral order,” in contrast to the present, characterized by “distrust” and “moral decay.”

Returning to Bahar’s narrative, in which she expressed strong regret concerning the decision to move to Vienna, we can still find positive and nostalgic representations of the city. Regardless of the difficult time Bahar experienced and her negative encounters with her children’s school, at times her narrative of her past life shifted from one of hardship to one of nostalgia and she even said with a sigh “I wish the good old days would return,” referring to the good times she had in Vienna. This came as quite a surprise considering the detailed description of the hardship after her and her children’s arrival in Vienna she narrated to me. This shift took place mostly when she explicitly compared present to past Vienna and her present to her past life. She fondly remembers the past in Vienna as the time when her children were young, she was in good health/shape and spent many happy hours with neighbors and their children in nearby parks. These snapshots of the good life in Vienna interrupted and contrasted with her elaborations on what she came to see retrospectively as her regrettable decision to move with her children to Vienna. The other time Bahar talked positively about Vienna was when she compared present Vienna to how it used to be. Then present Vienna became a dirty and an insecure place:

Well it is not like it used to be here. When we arrived it was really … I mean everything was so clean, the streets were so clean. We went out and came home late at night, at 1 or 2 am and nothing ever happened to us, not one incident. It was so clean and there were not yet so many different people [migrants of different nationalities], only Yugoslavs and Turks and there were only a few of them when we arrived. But now Vienna has really changed for the worse.

Such nostalgic discourses of past Vienna, regarding her present life and the wider societal situation including an increase in newcomers, reappeared several times during the two interviews and other conversations I had with her. Bahar did not attempt to explain this ambivalence to me; rather, the two narratives—of her hardships and disappointments as well as the good times she spent with her children in Vienna and the welcoming city she experienced—coexisted peacefully. I suggest we can speak of ambivalent nostalgia (Hirsch and Spitzer, 2002) here. As Hirsch and Spitzer show, conflicting memories may coexist without being reconciled (Hirsch and Spitzer, 2002: 260); that is, positive nostalgic feelings and negative ones, as in Bahar’s case, are not perceived as contradictory, but rather they simply coexist. Ambivalent feelings here are not perceived as contradictions but they represent two stances, one and the same person takes, without questioning their incompatibility.

In this regard, ambivalence comes to the fore in the phenomenon of nostalgia—particularly nostalgia for a lost home. Sometimes this was the old home in Turkey where one grew up, a home that no longer exists in the way it is remembered, not least because social relations changed in the meantime. Sometimes nostalgia was also directed toward past Vienna. Sometimes the lost home, however, was not geographically fixed but simply represented a safe place where moral order (still) prevailed. In these cases, nostalgic feelings directed toward Turkey or Austria or to a geographically non-specific place in the past often represented a critique of contemporary life and sometimes also of limited future prospects (see also Boyer, 2006; Palmberger, 2008, 2016). Men in particular made a stark contrast between their past and present lives in Austria. While they were welcomed as valuable labor migrants when they first came to Austria, with increasing age they experienced more and more discrimination, and not only in their workplaces. At the end of their careers and after retirement, they sense that the majority population increasingly sees them primarily as *Ausländer* (foreigners), and the fact that they had worked hard and also paid taxes, thereby contributing to Austria’s economic success, is no longer appreciated. They often set themselves apart from newly arrived “foreigners.” They linked the hostility they experienced to dominant discourses of Islamophobia in Austria, as well as anti-immigrant resentment and discrimination, including insulting statements from passers-by, passengers on public transport who refused to let them sit next to them or discrimination by the police.

## Assessing prospects for late life and care

On retirement and when children moved out of their parents’ home, for the first time it became possible to consider returning to Turkey. It was then that they traded off the positive against the negative aspects of living in Vienna. Approaching retirement age or recently retired, some contemplated whether to once again choose Turkey for their main residence.

Thoughts on whether to keep their main residence in Austria or return to Turkey are not necessarily tied to questions of where to call home because, as described above, often both places are given “home status,” albeit with different qualities. The issue is rather how to divide time between Turkey and Austria and consequently which of the two countries to choose as their main residence. Here structural constraints need to be considered. Several of the people I interviewed had no job and/or had applied for early retirement due to health issues. Being unemployed (and not yet granted retirement) restricts their mobility greatly. But also those retirees entitled to a small pension only receive payments when they remain based in Austria and they can spend a maximum of 8 weeks a year in another country. This conflicts greatly with their desire to spend a good part of the year in Turkey when retired. Preferences regarding mobility were central to their understandings of a satisfactory old age but were also significantly restricted by existing mobility regimes (Glick Schiller and Salazar, 2013).

In respect to the question of where to spend late life, it must be said that significant gender differences come to the fore, with men and women differing in their preferences. Conflicting ideas and dreams between couples often arise when preparing for late life. Orhan, whom I introduced above, experiences a similar conflict to Ahmet’s when it comes to his feelings of home and about where to spend (late) life compared with those of his wife and children:

If you ask me about home, honestly, I have to tell you that it is Turkey. But I have been here for 35 years and my wife was even born here. And so we have to stay here. I have two children—one is a trained psychologist, the other is studying tourism. We can’t leave Austria … you could say, our first country is Austria and our second is Turkey. But when you ask me about my home country, it is Turkey; it will never be Austria. But when you ask my wife, then she’ll say Austria is her home country. I just asked my son if he wants to come with me to Turkey since we have property there. I asked him if he wants to open a hotel there and he said “No Dad, I can’t live there; I live in Austria.”

While Orhan is not himself ambivalent about where he wants to spend late life, his feelings starkly contradict those of his other close family members, which causes a certain tension in their relationships and in their visions of a shared future. Orhan’s case is unusual since not only his children were born in Vienna but also his wife, whose parents migrated from Turkey to Vienna shortly before she was born. For Orhan, as for others, in the end the decision about how to share time between Turkey and Austria is made in dialogue with spouses and children. In interviews, this is sometimes explicit and at other times it takes the form of an inner dialogue in which relational ambivalence is prominent and the positions of other family members are represented.

Even if women expressed ambivalent feelings toward Vienna, as outlined above, they still preferred to spend the better part of their late life with their children and grandchildren there. But at the same time, they wanted to travel to Turkey regularly as long as they were physically able or at least to have an extended stay (up to a couple of months a year) in Turkey, preferably during summer time. Vienna had become the place where they had the strongest social networks, not only with family members but also with friends and sometimes neighbors. In Turkey, they were no longer integrated in everyday social activities and networks as Alevi, one of my female research participants, explained to me when she said, “I like it here [Austria] and there [Turkey]. I like it here; here I go to the mosque; I socialize. There, in Turkey, everyone is busy with their own lives … here in [Austria] I feel more at home.”

A crucial reason for feeling at home in Vienna that my female interlocutors voiced was that it was the place where their children and grandchildren lived. As other scholars observed among female labor migrants (Baldassar and Gabaccia, 2011; Ganga, 2006; Maynard et al., 2008; Zontini, 2015), the Turkish labor migrants too saw it crucial for their life quality to stay closely connected, also geographically, with children and grandchildren. This does not mean, however, that they did not from time to time secretly wish to be freed from being the family’s main care provider. For example, Bahar proudly presented herself as the person in the family who everyone shared their worries with and she saw it as her responsibility to help everyone. However, at times, she experienced this role as a burden she wanted to escape, at least for a while, and she dreamed of a journey to Mecca together with her female friends, which she eventually realized. While here we can speak of relational ambivalence, it is also a particularly gendered form of ambivalence. Connidis and McMullin (2002) relate this gendered nature of ambivalence to caregiving and to the structured relation between men and women that determines their share of care work. Women, who on a structural level are still expected to do the larger proportion of care work, may have less means to resist this pressure. Moreover, women are expected not only to do the greater share of care work but also to find it fulfilling. The ambivalent feelings this is likely to create are thus structurally inflicted (see Hochschild, 1997).

While the choice to keep their main residence in Austria was almost non-negotiable for women, some of my male interlocutors, in contrast, stated that they would prefer to return to Turkey and commute regularly to Vienna. They tended to have stronger ties to their hometown and seemed more integrated in social activities there, including with friends and family. As explained above, women remained the prime carers, looking after children (and grandchildren), including those that moved out long ago. Even if men became increasingly involved in emotional and care work after retirement, they still considered the option of returning to Turkey (at least for longer periods). Another crucial reason for men’s stronger ties to their hometown may be that the family house in Turkey was almost always located in the place where they grew up and where their parents lived, if still alive.

These differing preferences regarding where to spend late life often led to tensions within couples, as exemplified by Bartu and his wife. Since Bartu fell off a ladder in 2008 at the construction site he worked at, he has found it difficult to find a job and needs medical treatment, including surgery. He asked for invalid retirement but so far has been unsuccessful. Bartu told me that he misses Turkey a great deal and hopes to return in the near future. He asked his wife to return with him, especially after all the troubles he went through with temporary employment agencies, which he said were particularly cruel for a person of his age. Bartu’s wife, however, was unwilling to return with him, and instead she insisted that he remain with her in Austria and their two children and nine grandchildren. This is the answer Bartu gave me when I asked him about his post-retirement plans:

Yes, I want to return … because you want to live in the place you were born in. I am not saying that Austria is bad; no, it is very good … I like it here; it is an easier life here than in Turkey … for example public transport … but still, you long for your place of birth, right?

Bartu describes life in Vienna as more comfortable but life in Turkey as “his” life, in a place in which he still feels he belongs. Yet, at the end of his explanation, he seeks reassurance when he says, “right?” After a while he continued to explain his difficult situation “in between” two countries—Turkey, his country of birth and the country his parents still live in, and Austria, the country he has spent most of his life in and where his children were born and feel at home. His words exemplify the ambivalent feelings of belonging I so often encountered:

We can’t return to Turkey … the children are here. Actually I wanted to move back for good this year but I couldn’t convince my wife. I wanted to return to Turkey because here the employment center won’t leave me in peace … when you are off sick, the health insurance bothers you … then they connect you with agencies that offer temporary jobs—that has become a problem! And because I am old, companies no longer want to employ me. They would employ the young ones but they don’t want to work anyway … for that reason we can’t return [to Turkey]. In the end I agreed with my wife to stay here, because our children live here. But I am worried because my mother and father live in Turkey. They got sick and need care. But if we were to leave, our children would also worry about us all the time; that is also a problem … but we will probably stay here.

Here Bartu’s relatives become central to his decision-making process: both parties (his children and parents) are present, and he seems to want to do justice to both. And then there is the conflict between his desire to return and his wife’s to keep their main residence in Austria. As becomes clear in Bartu’s case, while individuals have ambivalent feelings in regard to their new and old homes, married couples also need to negotiate conflicting interests, most often resulting in a compromise. This corresponds with research with aging labor migrants in other European countries. Zontini (2015), for example, shows in the case of aging Italian labor migrants in the United Kingdom that gender is crucial in analyzing the elders’ experiences. Bolzman et al. (2006) also show in the case of Italian and Spanish labor migrants in Switzerland how gender discrepancies become prominent when questions of return are negotiated (see also Bolzman et al. 2017). Despite gender differences, most couples in the end opted for one extended stay in Turkey, from 1 to 3 months (preferably during/including summertime) and spending the rest of the year with their children (and grandchildren) in Vienna. Thus, the question of where to spend late life should not be understood as a one-time decision but rather as a process, which some authors claim to be intrinsic to the aging migrant experience (see Ganga, 2006). This also means that a clear decision may never be made or that plans to return may never be realized.^2^

My interlocutors often found the subject of late-life care in the event of physical and mental health problems hard to address. It also became clear that families did not openly discuss questions of late-life care. This was especially noticeable when the children were present during informal interviews. Many of the older migrants, it has to be added, still perceive themselves as care “providers” since they take on a good share of daily care activities, including looking after grandchildren but also adult children with a disability or chronic illness, and it is not unusual for mothers to still cook for their adult children when the latter have busy working lives. When I asked my interviewees about where and from whom they would seek help if they were in need for care, most of them said they hoped their children would take care of them when they needed it. However, they are mostly aware that they could not take this help for granted and they said, “times have changed.”

They know that the young generation are busy with work and raising children and only have a little time left to care for older family members. They added that they would seek institutional care if necessary. Women usually stated that they would look for an institution in Austria first, while men sometimes preferred a place in their country of origin. Both men and women, however, in the end are highly ambivalent about whether an old people’s home in Austria would satisfy their needs (Palmberger, 2018). Language difficulties were always raised as an issue. Moreover, questions of faith, such as provision of prayer rooms and food, especially the availability of halal options, are also important to many interviewees. These specific ideas and desires for late life that my interviewees articulated support the argument that “feelings of belonging change over the life course in complex ways” (Zontini, 2015: 338). It is thus crucial to bear changing life situations in mind—such as the post-retirement phase—when analyzing migrants’ lifeworlds. This is also the case when researching transnational connections, which require a life course approach (see King et al., 2006).

## Conclusion

By introducing the concept of *relational ambivalence* in this article, I have shown that ambivalence can neither be seen solely as an individual experience, as residing within the individual, nor as a result of differing social status and roles alone. Rather it is a product of relationships individuals engage in. This also means that relational ambivalence can be marked by ambivalences stemming from gender structures. Offering such a relational approach to ambivalence adds an important angle of analysis that takes migrants’ interpersonal relations and life course perspectives seriously.

My findings on relational ambivalence among former guest workers, based on a narrative and life course approach, reveal that retirement is an important phase in the life of migrants as it is a time of reflection on their migration history and of decision-making for late life. More specifically, this period is characterized by multiple ambivalences, which come to surface most clearly when past choices (particularly the decision to migrate to Austria), questions of belonging and such future decisions as where to spend late life and how to organize late-life care are tackled. Emotions are central—not only the individual’s but also those of close family members—in the migrants’ decision-making, often triggering ambivalent feelings. Spouses and children and their specific needs and desires influence the decision-making process significantly. When my interlocutors weighed pros and cons, it seemed that this was not done to convince me, the interviewer, but rather in conversation with several voices (particularly of family members) and arguments were brought up in response to different addressees. The narratives I encountered always represented several (often seemingly opposing) views and standpoints and they often resembled an inner dialogue that seemed ongoing from long before the actual conversation took place. It is in this sense that ambivalence must be understood as a product of relationships rather than solely an individual experience. The concept of relational ambivalence captures these social and discursive dimensions of ambivalence.

Rather than accepting ambivalences as contradictions in the first instance, I suggest drawing attention to the specific case and to the speaker’s own interpretation, which may reveal ambivalences to be more or less disturbing, or sometimes that ambivalent positions simply coexist peacefully. At times, ambivalence is even linked to hope, thus granting it a future-oriented component. Hague (2016) discusses ambivalence in relation to hope and faith in the future when he describes ambivalence and uncertainty as the core of hope. Even when individuals find themselves in a state of uncertainty, they may remain hopeful at the same time. Thus, being “caught up” in ambivalent feelings and positions does not necessarily mean having a pessimistic view of the future. Similarly, I suggest that indecision or the indeterminate deferral of decisions does not represent a passive response but may well be a deliberate strategy when confronted with seemingly irresolvable life situations. With this, the article attempts to contribute to a more differentiated debate on ambivalence, one that takes the emotional and social dimension of ambivalence seriously in migratory contexts and beyond.

Even though I showed in this article how decision-making processes and reflections on the past are a shared endeavor (rather than an individualistic project), I analyzed ambivalences against the backdrop of different structural constraints and exclusion mechanisms, which strongly affected my interviewees’ and their family’s life. Exclusion and resentments concerned, that is, educational discrimination (in case of labor migrants’ children) and discrimination labor migrants experienced at the end of their working life and once they had retired. In the post-retirement phase, structural constraints often imply restricted geographical mobility that conflict with migrants’ commuting preferences. Furthermore, aging labor migrants face difficulties in access to institutionally based elder care due to a lack of cultural-sensitive care. These structural constraints strongly affect family life and decision-making for late life. They ultimately limit individuals’ choices in old age. These findings have important implications for policy makers, in Austria and beyond. A focus on relational ambivalence that incorporates broader societal structures can provide essential insights into inclusion/exclusion mechanisms that affect migrants’ lives, also and particularly during old age.

## Notes

1. Merriam-Webster’s dictionary, https://www.merriam-webster.com/dictionary/ambivalence.

2. With the exception of the majority of my Alevi research participants (who did not have a positive relationship with present-day Turkey and its politics) all of my interviewees—men and women alike—wished to be buried in Turkey when the time came.

## References

[bibr1-0020715219832918] Abu-Lughod L Lutz CA (1990) Introduction: Emotion, discourse, and the politics of everyday life. In: Abu-Lughod L Lutz CA (eds) Language and the Politics of Emotion. Cambridge: Cambridge University Press, pp. 1–23.

[bibr2-0020715219832918] Ahmed S (1999) Home and away: Narratives of migration and estrangement. International Journal of Cultural Studies 2(3): 329–347.

[bibr3-0020715219832918] Baldassar L Gabaccia DR (2011) Intimacy and Italian Migration: Gender and Domestic Lives in a Mobile World. New York: Fordham University Press.

[bibr4-0020715219832918] Bauman Z (1990) Modernity and ambivalence. Theory, Culture & Society 7(2–3): 143–169.

[bibr5-0020715219832918] Baykara-Krumme H (2014) Returning, staying, or both? Mobility patterns among elderly Turkish migrants after retirement. Transnational Social Review 3(1): 11–29.

[bibr6-0020715219832918] Berliner D (2016) Anthropology and the study of contradictions. Hau: Journal of Ethnographic Theory 6(1): 1–6.

[bibr7-0020715219832918] Bilecen B (2017) Home-making practices and social protection: An example of Turkish migrants living in Germany. Journal of Housing and Built Environment 32: 77–90.

[bibr8-0020715219832918] Bilecen B Catir G Orhon A (2015) Turkish-German transnational social space: Stitching across borders. Population, Space and Place 21: 244–256.

[bibr9-0020715219832918] Boccagni P (2012) Rethinking transnational studies: Transnational ties and the transnationalism of everyday life. European Journal of Social Theory 15(1): 117–132.

[bibr10-0020715219832918] Boccagni P (2017) Migration and the Search for Home. Mapping Domestic Space in Migrants’ Everyday Lives. Basingstoke: Palgrave Macmillan.

[bibr11-0020715219832918] Boccagni P Baldassar L (2015) Emotions on the move: Mapping the emerging fields of emotion and migration. Emotion, Space and Society 16: 73–80.

[bibr12-0020715219832918] Bolzman C Kaeser L Christe E (2017) Transnational mobilities as a way of life among older migrants from Southern Europe. Population, Space and Place 23(5): 73–80.

[bibr13-0020715219832918] Bolzman C Fibbi R Vial M (2006) What to do after retirement? Elderly migrants and the question of return. Journal of Ethnic and Migration Studies 32(8): 1359–1375.

[bibr14-0020715219832918] Boyer D (2006) Ostalgie and the politics of the future in Eastern Europe. Public Culture 18(2): 361–382.

[bibr15-0020715219832918] Ciubanu O Nedelcu M (2019) Ageing as a Migrant: Vulnerabilities, Agency and Policy Implications. London and New York: Routledge.

[bibr16-0020715219832918] Cohen L (1994) Old age: Cultural and critical perspectives. Annual Review of Anthropology 23: 137–158.

[bibr17-0020715219832918] Connidis I McMullin J (2002) Sociological ambivalence and family ties. Journal of Marriage and the Family 64(3): 558–567.

[bibr18-0020715219832918] Dwyer C (2000) Negotiating diasporic identities: Young British South Asian Muslim women. Women’s Studies International Forum 23(4): 475–486.

[bibr19-0020715219832918] Ehrkamp P (2005) Placing identities: Transnational practices and local attachments of Turkish immigrants in Germany 31(2): 345–364.

[bibr20-0020715219832918] Ganga D (2006) From potential returnees into settlers: Nottingham’s older Italians. Journal of Ethnic and Migration Studies 32(8): 1395–1413.

[bibr21-0020715219832918] Giddens A (1994) Living in a post-traditional society. In: Beck U Giddens A Lash S (eds) Reflexive Modernization: Politics, Tradition and Aesthetics in the Modern Social Order. Cambridge: Polity Press, pp. 56–109.

[bibr22-0020715219832918] Glick Schiller N Caglar A (2011) Locating Migration: Rescaling Cities and Migrants. Ithaca, NY; London: Cornell University Press; Journal of Ethnic and Migration Studies.

[bibr23-0020715219832918] Glick Schiller N Salazar N (2013) Regimes of mobility across the globe. Journal of Ethnic and Migration Studies 39(2): 183–200.

[bibr24-0020715219832918] Hague G (2016) Questions concerning a future-politics. History and Anthropology 27(4): 465–467.

[bibr25-0020715219832918] Hillcoat-Nalletamby S Philipps JE (2011) Sociological ambivalence revisited. Sociology 45(2): 202–217.

[bibr26-0020715219832918] Hirsch M Spitzer L (2002) We would not have come without you: Generations of nostalgia. American Imago 59(3): 253–276.

[bibr27-0020715219832918] Hochschild A (1983) The Managed Heart: Commercialization of Human Feeling. Berkeley, CA: University of California Press.

[bibr28-0020715219832918] Hochschild A (1997) The Time Bind: When Work Becomes Home and Home Becomes Work. New York: Metropolitan.

[bibr29-0020715219832918] Hromadzic A Palmberger M (2018) Care across Distance: Ethnographic Explorations of Aging and Migration. Oxford and Brooklyn, NY: Berghahn Books.

[bibr30-0020715219832918] Jovanovic D (2016) Ambivalence and the study of contradictions. Hau: Journal of Ethnographic Theory 6(1): 1–6.

[bibr31-0020715219832918] Karl U Torres S (eds) (2016) Ageing in Contexts of Migration. Oxon and New York: Routledge.

[bibr32-0020715219832918] Keith J (1980) “The best is yet to be.” Toward an anthropology of age. Annual Review of Anthropology 9: 339–364.

[bibr33-0020715219832918] Kelle U (2007) The development of categories: Different approaches in grounded theory. In: Bryant A Charmaz K (eds) The Sage Handbook of Grounded Theory. Los Angeles, CA: SAGE, pp. 191–213.

[bibr34-0020715219832918] Kierans C Bell K (2017) Cultivating ambivalence. Some methodological considerations for anthropology. Hau: Journal of Ethnographic Theory 6(1): 23–44.

[bibr35-0020715219832918] King R Dalipaj M Mai N (2006) Gendering migration and remittances: Evidence from London and Northern Albania. Population, Space and Place 12(6): 409–434.

[bibr36-0020715219832918] Kivisto P La Vecchia-Mikkola V (2013) Immigrant ambivalence toward the homeland: The case of Iraqis in Helsinki and Rome. Journal of Immigrant & Refugee Studies 11(2): 198–216.

[bibr37-0020715219832918] Lambek M (2016) On contradictions. Hau: Journal of Ethnographic Theory 6(1): 6–8.

[bibr38-0020715219832918] Leavitt J (1996) Meaning and feeling in the anthropology of emotions. American Ethnologist 23(3): 514–539.

[bibr39-0020715219832918] Maynard MA Afsher H Franks M et al (2008) Women in Later Life: Exploring Race and Ethnicity. Maidenhead: Open University Press.

[bibr40-0020715219832918] Merton RK (1976) Sociological Ambivalence and Other Essays. New York: Free Press.

[bibr41-0020715219832918] Palmberger M (2008) Nostalgia matters: Nostalgia for Yugoslavia as potential vision for a better future. Sociologija 50(4): 355–370.

[bibr42-0020715219832918] Palmberger M (2016) Social ties and embeddedness in old age: Older labour migrants in Vienna. Journal of Ethnic and Migration Studies 43(2): 235–249. 28392746 10.1080/1369183X.2016.1238907 PMC5359779

[bibr43-0020715219832918] Palmberger M (2018) Social embeddedness and care among Turkish labor migrants in Vienna: The role of migrant associations. In: Hromadzic A Palmberger M (eds) Care across Distance: Ethnographic Explorations of Aging and Migration. Oxford and Brooklyn, NY: Berghahn, pp. 97–112.

[bibr44-0020715219832918] Palmberger M Gingrich A (2013) Qualitative comparative practices: Dimensions, cases, and strategies. In: Flick U (ed.) Sage Handbook of Analyzing Qualitative Data. Thousand Oaks, CA: SAGE, pp. 94–108.

[bibr45-0020715219832918] Palmberger M Hromadzic A (2018) Introduction: Care across distance. In: Hromadzic A Palmberger M (eds) Care across Distance: Ethnographic Explorations of Aging and Migration. Oxford and Brooklyn, NY: Berghahn, pp. 1–12.

[bibr46-0020715219832918] Peletz M (2001) Ambivalence in kinship since the 1940s. In:Franklin S McKinnon S (eds) Relative Value. Durham: Duke University Press, pp. 413–442.

[bibr47-0020715219832918] Reinprecht C (2006) Nach Der Gastarbeit: Prekäres Altern in Der Einwanderungsgesellschaft. Vienna: Braumüller.

[bibr48-0020715219832918] Smart B (1999) Facing Modernity: Ambivalence, Reflexivity and Morality. London: SAGE.

[bibr49-0020715219832918] Smelser NJ (1998) The rational and the ambivalent in the social sciences. American Sociological Review 63(1): 1–16.

[bibr50-0020715219832918] Uehling G (2002) Sitting on suitcases: Ambivalence and ambiguity in the migration intentions of Crimean Tatar women. Journal of Refugee Studies 15(4): 388–408.

[bibr51-0020715219832918] Vertovec S (2004) Migrant transnationalism and modes of transformation. International Migration Review 38(3): 970–1001.

[bibr52-0020715219832918] Von Lorber V (2017) Angeworben. Gastarbeierinnen in Österreich in Den 1960Er Und 1970er Jahren. Göttingen: V&R Unipress.

[bibr53-0020715219832918] Weigert A (1991) Mixed Emotions: Certain Steps Toward Understanding Ambivalence. Albany, NY: State University of New York Press.

[bibr54-0020715219832918] Williams R (2009) On structure of feeling. In: Harding J Pribram E (eds) Emotions: A Cultural Studies Reader. London; New York: Routledge, pp. 35–49.

[bibr55-0020715219832918] Wong L (2002) “Home away from home?” Transnationalism and the Canadian citizenship regime. In: Kennedy P Roudometof V (eds) Communities across Borders: New Immigrants and Transnational Cultures. London; New York: Routledge, pp. 169–181.

[bibr56-0020715219832918] Yeoh B Huang S (2000) “Home” and “away”: Foreign domestic workers and negotiations of diasporic identity in Singapore. Women’s Studies International Forum 23(4): 413–429.

[bibr57-0020715219832918] Zontini E (2015) Growing old in a transnational social field: Belonging, mobility and identity among Italian migrants. Ethnic and Racial Studies 38(2): 326–341.

